# What limits the performance of current invasive brain machine interfaces?

**DOI:** 10.3389/fnsys.2014.00068

**Published:** 2014-04-29

**Authors:** Gytis Baranauskas

**Affiliations:** Neurophysiology Laboratory, Neuroscience Institute, Lithuanian University of Health SciencesKaunas, Lithuania

**Keywords:** brain machine interface, brain computer interface, extracellular recordings, information, throughput, multichannel recordings

## Abstract

The concept of a brain-machine interface (BMI) or a computer-brain interface is simple: BMI creates a communication pathway for a direct control by brain of an external device. In reality BMIs are very complex devices and only recently the increase in computing power of microprocessors enabled a boom in BMI research that continues almost unabated to this date, the high point being the insertion of electrode arrays into the brains of 5 human patients in a clinical trial run by Cyberkinetics with few other clinical tests still in progress. Meanwhile several EEG-based BMI devices (non-invasive BMIs) were launched commercially. Modern electronics and dry electrode technology made possible to drive the cost of some of these devices below few hundred dollars. However, the initial excitement of the direct control by brain waves of a computer or other equipment is dampened by large efforts required for learning, high error rates and slow response speed. All these problems are directly related to low information transfer rates typical for such EEG-based BMIs. In invasive BMIs employing multiple electrodes inserted into the brain one may expect much higher information transfer rates than in EEG-based BMIs because, in theory, each electrode provides an independent information channel. However, although invasive BMIs require more expensive equipment and have ethical problems related to the need to insert electrodes in the live brain, such financial and ethical costs are often not offset by a dramatic improvement in the information transfer rate. Thus the main topic of this review is why in invasive BMIs an apparently much larger information content obtained with multiple extracellular electrodes does not translate into much higher rates of information transfer? This paper explores possible answers to this question by concluding that more research on what movement parameters are encoded by neurons in motor cortex is needed before we can enjoy the next generation BMIs.

Although the idea of a direct control of devices by human mind can be tracked down to the first experiments relating brain signals to behavior (Humphrey et al., 1970; Kennedy et al., 1992; Kennedy and Bakay, 1998; Wolpaw et al., 2002; Mussa-Ivaldi and Miller, 2003), only the advent of more powerful computer technologies in the last few decades enabled routine testing of these ideas in the environment of a scientific laboratory and, in some cases, in real life (Chapin et al., 1999; Taylor et al., 2002; Carmena et al., 2003; Velliste et al., 2008). This lead to the use of term “brain machine interface” (BMI) for such devices and in the last 15 years the number of papers published per year in the field of BMI increased exponentially (Figure 1). During this period it has been demonstrated that not only rats (Chapin et al., 1999) and monkeys (Wessberg et al., 2000) but also humans can control both a computer cursor and a prosthetic arm by their brain activity (Kennedy et al., 2000; Wolpaw and McFarland, 2004; Hochberg et al., 2006, 2012; Kim et al., 2008). Although already in late '90s Kennedy and his colleagues employed a single electrode in human patients to conditionally control activity of single units (Kennedy and Bakay, 1998) and then a computer cursor (Kennedy et al., 2000), usually only experiments with multiple electrodes are considered to be relevant to BMIs of today and the tests with a robotic lever in rats are viewed as the birth of the modern BMI concept (Chapin et al., 1999; Lebedev and Nicolelis, 2006). Thus for electrode arrays it is possible to claim that in less than a decade the idea of BMI made a jump from the animal research to the tests in humans (Hochberg et al., 2006), a spectacular achievement.

**Figure 1:**
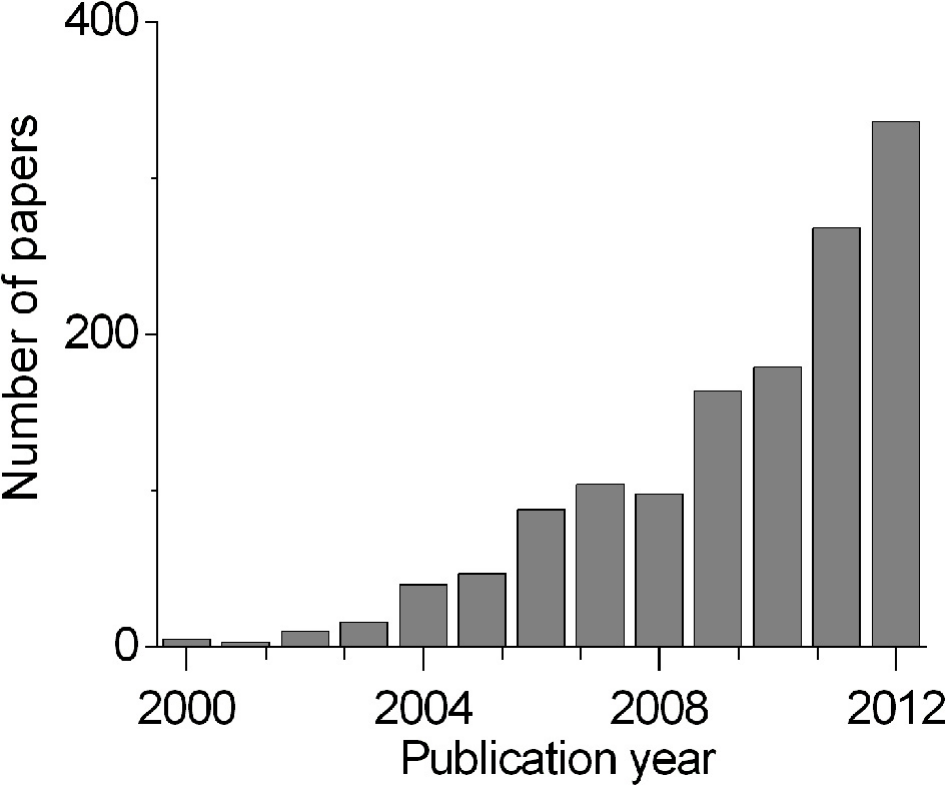
**The number of papers published each year with terms “Brain Machine Interface,” PubMed search data**.

However, did we achieve a qualitative improvement in invasive BMIs? This paper tries to answer this question by applying a single measure- information transfer rate. Although it may be viewed as a rather narrow-minded approach to evaluate the progress in a large field of science and technology by applying a single measure, the author believes that such a unified approach can be very useful in determining the strategy for future development of research and technology. This view is shared by a number of researchers as attested by a recent focused review on the topic (Tehovnik et al., 2013), a few reviews on EEG-based non-invasive BMIs (Wolpaw et al., 2000, 2002; Haselager, 2013), some papers on invasive BMIs (Gilja et al., 2012) and the position taken by the Defense Advanced Research Project Agency (DARPA), which funded many outstanding labs in the field of invasive BMI during the last few decades (Judy, 2012). The agency states that higher information transfer rates and increased durability/stability of BMI devices are the primary goals for this field of research (Judy, 2012). The information transfer rate is directly related to the ability to control a device (Tonet et al., 2008; Haselager, 2013; also see below) and may not be easily applied to some recently proposed new applications of BMIs such as restoration of neuronal function (Grosse-Wentrup et al., 2011). Nevertheless, device control remains the mainstream of BMI research and in these applications the information transfer rates can be used as the main quantitative measure to evaluate the overall system performance (Tonet et al., 2008; Haselager, 2013; Tehovnik et al., 2013).

Originally the mathematical information theory was developed to help optimize the communication line capacity (Shannon, 1948); these lines were mainly used for telegraph at that time, however, the theory is sufficiently general to be applied to any communication line, including the one used for a device control in BMI and the theory provides a quantitative measure, information content, that is independent of how BMI functions.

Even though to quantify BMI performance we employ just a single measure, the information transfer rate, we are still facing a daunting task to estimate information content in behavioral tasks that are used for BMI capability demonstration. The mathematical information theory determines what it takes to reproduce “at one point either exactly or approximately a message selected at another point” in space and time (Shannon, 1948). Shannon talks about a message selected from a set of messages and it is not important what is actually written in the message, the only thing that matters is how many such messages can be chosen, the number of possible selections. In a center-out reaching behavioral task, which is frequently used for BMI demonstrations (Taylor et al., 2002; Wolpaw and McFarland, 2004; Mulliken et al., 2008) and in which typically a subject has to choose several targets on the screen by moving a cursor from a central location, one message can correspond to one target. If during all runs 8 different targets were presented, we have 8 different messages. The information content is measured as a logarithm of number of choices to base of 2; for 8 messages with an equal probability to be presented the information content will be log_2_(8) = 3. By taking into account the error rates and the time needed to accomplish the task one can obtain the information transfer rate values (Tonet et al., 2008; Tehovnik et al., 2013). However, this approach has caveats and such a simple information content estimate is insufficient when we evaluate BMI performance. In this task the target relative size affects the subject performance and the larger target size will lead to higher information transfer rate even though no actual change in BMI performance will be present. The question of such task difficulty has been investigated in a number of papers on human-computer interactions (Fitts, 1954; MacKenzie and Buxton, 1992) and the Fitts' law is probably the best for evaluation of information content in such tasks. For other types of tasks, such as reaching and grasping (Carmena et al., 2003), the parameters determining task difficulty can be only guessed and there is a need of experimental data that information content of such tasks is evaluated. As long as no such experimental data exist, we are limited to these BMI studies that employ center-out reaching task. Luckily, this behavioral task is, probably, the most frequently used behavioral task in invasive BMI research from early on and even by limiting analysis to the center-out reaching task a trend in invasive BMI performance can be detected and few conclusions made.

In this review the author will try to show that the fundamental issues of biology but not computer power or electronic circuit capabilities are the limiting factors in achieving higher information transfer rates in invasive BMIs of today. More specifically, the author believes that, to increase invasive BMI performance, a more profound understanding about what neurons encode in motor areas of our brain is necessary and, actually, such BMIs can be a testing ground for new ideas on what kind of information is present in neuronal signals.

The paper is organized as follows. First, differences between invasive and non-invasive BMIs will be explained in brief. Second, the relationship between the information transfer rates and practical applications is discussed; examples from both invasive and non-invasive BMI will be presented. Third, technological factors that affect information rates in invasive BMIs will be evaluated. Finally, two examples of high information transfer rates in invasive BMIs will be analyzed and the strategies to improve information transfer rates will be suggested.

Today the term BMI can be applied to very different devices, from head caps with electrodes for electro-encephalogram (EEG) recording, in which EEG signal is transformed to commands for letter selection on a computer screen (Wolpaw et al., 2002; Krusienski et al., 2008) to implanted electrode arrays in monkey or, less frequently, rat and human brains, enabling a robotic arm control (Hochberg et al., 2006; Nicolelis and Lebedev, 2009). Since there are vast technological differences between a head-cap with electrodes for EEG and an array of wires inserted into the brain to record single neurons or field potentials of neuronal assemblies, usually all BMIs are broadly divided into two groups (Wolpaw et al., 2002; Tonet et al., 2008):

1. Non-invasive BMIs, mainly EEG-based, although there are also systems that use muscle signals (EMG), gaze direction (Surakka et al., 2004; Oskoei and Hu, 2007; Tuisku et al., 2012) and other signals that do not require a surgical intervention.
2. Invasive BMIs, these systems require a surgical intervention for electrode insertion and include not only electrode arrays inserted into the cortex but also electrodes implanted into the body for peripheral nerve activity detection (Navarro et al., 2005) or any other type of BMI that require extensive surgical procedure, for instance, cochlear implants (Moller, 2006).

These two classes of BMIs can be separated not only on the basis of technological differences but also on the basis of ethical problems associated with surgery in invasive BMIs. Invasive BMIs always require at least some surgery, which could be painful and, possibly, risky and there is always a question if such risks are offset by the benefits of BMI. In addition, invasive BMIs are usually much more expensive than non-invasive ones. Therefore, the expectation bar for invasive BMIs is higher than for non-invasive BMIs.

In spite of differences, all BMIs can be defined as devices that process information detected in the brain activity; the extracted information is used to determine the subject's intent and to control a computer cursor or a prosthesis. Thus, from information theory point of view the key characteristic of such a device is the amount of information transferred per unit of time, or channel capacity if we use Shannon's terms (Shannon, 1948) or throughput as it is called in many recent papers (Tonet et al., 2008; Gilja et al., 2012). An attempt to take a unified approach in the BMI research dates back to at least the first international meeting on non-invasive BMIs (Wolpaw et al., 2000). However, the use of information transfer rates became more common in the field of invasive BMIs only recently (Simeral et al., 2011; Gilja et al., 2012; Flint et al., 2013). Thus it is not an accident that the mentioned above DARPA initiative aimed to advance significantly the development of the upper-limb prosthesis technology places the information transfer rates at the center of its stated goals. The main reason for such an emphasis on information transfer rates is that in the design of neuroprosthesis this rate determines the functionality of a device (Tonet et al., 2008; Haselager, 2013). To understand that, we can take a wheelchair controlled by BMI as an example. One of the most important features of a wheelchair is the ability to stop it in case of emergency. The information transfer rate can be directly translated into the time required for such a command, because the command has 1 bit of information. Since typical EEG-based BMIs have information transfer rates of 0.25–0.5 bits/s (Wolpaw et al., 2002; Allison et al., 2012), for a EEG-based BMI it will take at least 2–4 s to stop a wheelchair. This is hardly acceptable because even at a very moderate speed of 0.5–1 m/s the wheelchair will move 1–2 m before it will stop. Although for non-emergency cases one can anticipate when to stop but if something unexpected happens there is no time for preparation, thus at least two-fold faster information transfer rates are needed for efficient stopping of a wheelchair in case of emergency.

More complex behaviors such as an arm movement correspond to much higher information transfer rates. To give an example, Paul Fitts in his famous paper (Fitts, 1954) estimated that in a simple tapping task human subject routinely achieve ~10 bit/s information transfer rates. It is likely that the control of a robotic arm with many degrees of freedom will require even higher information transfer rates. Human speech can be used as another example of a typical information rate routinely achieved by our brains. In a slow human speech ~100 words are produced each minute, or 1–2 words per second. If we use word recognition perplexity to estimate information content of each word (Brown et al., 1992), then each English word contains ~7.5 bits of information on average, corresponding to 7–15 bit/s of information transfer rates in a slow human speech. These examples show that fluent interactions with human beings require information transfer rates of ≥~10 bit/s and, keeping in mind that information unit, a bit, is defined in a logarithmic scale, this information transfer rate is several orders of magnitude higher than achieved by most BMIs today, usually <3 bit/s (see below). It may be argued that some human patients could benefit even from such low information transfer rates (Wolpaw et al., 2002) but an example with a wheelchair shows that even for such patients increasing these rates is critical for at least some functions.

Lower information transfer rates mean less fun as it has been discovered by companies making EEG-based devices for entertainment. A number of devices are already on the market such as several toys made by Neurosky, Mindball made by Interactive Productline and few others. Although Neurosky claims that several of its toys had “a phenomenal success,” all these devices, according to their users, share one thing in common- they are difficult to control. More specifically, not only it takes time to learn to use them but also the achieved control is unreliable. Some users even claimed that actually no control was achieved by such devices, in many cases brain signals are of low quality because of the presence of artifacts (Fatourechi et al., 2007). Thus in spite of relatively low prices of some of these devices, the lowest being approximately 100$, the user experience is still somehow limited in spite of attractiveness of the idea to directly control a device by thought.

From the point view of information theory, unreliable control is equivalent to low information transfer rates. Today EEG-based BMIs are limited to information transfer rates of <0.5 bit/s (Klobassa et al., 2009; Townsend et al., 2010). To put this number in perspective, we can take as a benchmark information transfer rates achieved by human subjects in a simple motor task of tapping (Fitts, 1954) and in speech recognition employing cochlear implants, the first BMIs used in large numbers (Mussa-Ivaldi and Miller, 2003). The gap between the EEG-based BMI information transfer rate of 0.5 bit/s and the 10 bit/s rate achieved in both cochlear implants used for human speech recognition (Dunn et al., 2010), and the tapping task is frightening. Since the bit scale is logarithmic, a difference of 10 bits in information content corresponds to 1000 times higher information content. It is true that cochlear implants, so far the only invasive BMIs in wide use, started slowly in 50 and 60 s. The first users of cochlear implants were able to have only some comprehension of sounds but not speech, corresponding to very low information transfer rates, probably of the order of few bits per second (Moller, 2006). However, the ability to recognize human speech was, almost certainly, a key to their widespread use and today >40,000 (by some estimates >20,000) of these devices have been implanted (Rauschecker and Shannon, 2002).

These considerations should suffice to convince the reader that, at least to some degree, progress in the development of BMIs can be evaluated by looking at how much information transfer rates were improved over the years of research. In non-invasive BMIs there is a consensus that current limit is ~0.5 bit/s and it is largely unchanged in the last 10–15 years (Wolpaw et al., 2002; Krusienski et al., 2008; Townsend et al., 2010). This notion is less obvious for invasive BMIs. One problem in this field is that only recently the need for a uniform measure of information content has been more widely recognized and employed in the result description (Li et al., 2009; Simeral et al., 2011; Gilja et al., 2012; Flint et al., 2013). Although to calculate the information content of a behavioral task the information theory allows the use of almost any movement parameter such as the target number or the target coordinate (Tonet et al., 2008; Tehovnik et al., 2013), to have a meaningful comparison of BMI performance in different tasks, the comparison that permits to decide which algorithm, parameter choice or configuration of BMI is better, we need to take into account the difficulty of task. The concept of difficulty can be easily explained in a center-out reaching task (Taylor et al., 2002; Hochberg et al., 2006), in which a subject has to reach from the monitor center a target located some distance from the initial position. Although it is possible to take the logarithm of the number of targets as the information content of such a task, intuitively it seems obvious that larger targets are easier to reach. Fitts' studies for one-dimensional tasks of this type confirmed that target size but not number of targets determines how long it takes to reach the target (Fitts, 1954). He introduced an index of difficulty, defined as

$$I_{d}=-log_{2}\frac{W_{s}}{2A}\;bits/response,$$

where *W*_s_ is the target size (“tolerance range” in the original paper) and A is the average amplitude of movements, corresponding to the average distance to the target. Importantly, Fitts showed that for this measure of difficulty the limit of human performance was always the same, 10 bits/s, independent of target size, distance to the target etc. Thus, if this index of difficulty is used as the information content of the task, then differences in center-out task performance should indicate differences in the BMI performance but not the task difficulty. Although attempts were made to extend this method to 2D and 3D tasks (MacKenzie and Buxton, 1992), there is no consensus what difficulty measure should be used in such cases. Recently an alternative approach has been suggested, SNR, a logarithm of the ratio of the actual value variance to the mean squared error of the predicted values (Li et al., 2009). It is reminiscent of the index of difficulty, where target size is substituted by the mean square of the predicted values and the average amplitude of movements by the actual value variance. However, first it has to be demonstrated experimentally that SNR evaluates correctly the difficulty of a behavioral task. SNR is only one possible measure to evaluate BMI performance in motor tasks, one can invent many other measures and the question is what these measures tell us. If exactly the same task is performed by different BMIs, it is straightforward to compare the performance of these BMIs. However, if tasks differ even in a single parameter, be it target presentation time or movement speed, it is much more difficult to compare the performance of these BMIs. We need a measure that represents BMI performance in a task-independent manner, something what Fitts demonstrated for human performance in several different tasks: his difficulty measure returned similar performance values (<20% change) for movement distances covering more than one order of magnitude (Fitts, 1954). If SNR is an objective measure of BMI performance it should produce the same or similar value for all tasks performed by the same BMI; to verify this statement we need experimental data.

For a symmetrical center-out reaching task the above Fitts' formula can be easily applied because distance to all targets is the same. Therefore in this review the analysis of BMI performance is limited to a single behavioral task, the center-out reaching task. Luckily, a sufficiently large number of studies employed this task over the span of more than one decade and the results from probably the best laboratories in the field of BMI can be included (Figure 2). The results presented in Figure 2 show little or no improvement in the information transfer rates over ~10 years of research except for two data points, marked in red and black (Figure 2). These two outliers will be discussed below. If we neglect the outliers, the obvious question is why so much effort resulted in not much improvement in probably the main parameter determining the performance of invasive BMIs? A number of brilliant people were involved in this type of research and there must be a serious reason why we see little progress toward improvement in this key parameter. The next few paragraphs will be devoted to an attempt to answer this question. First the issues of technology will be very briefly explained, with a focus on the question if the current state of computer power, electrode technology impedes to achieve higher information transfer rates. Then an answer to the main question of this paper, how to improve the performance of invasive BMIs, will be sought.

**Figure 2:**
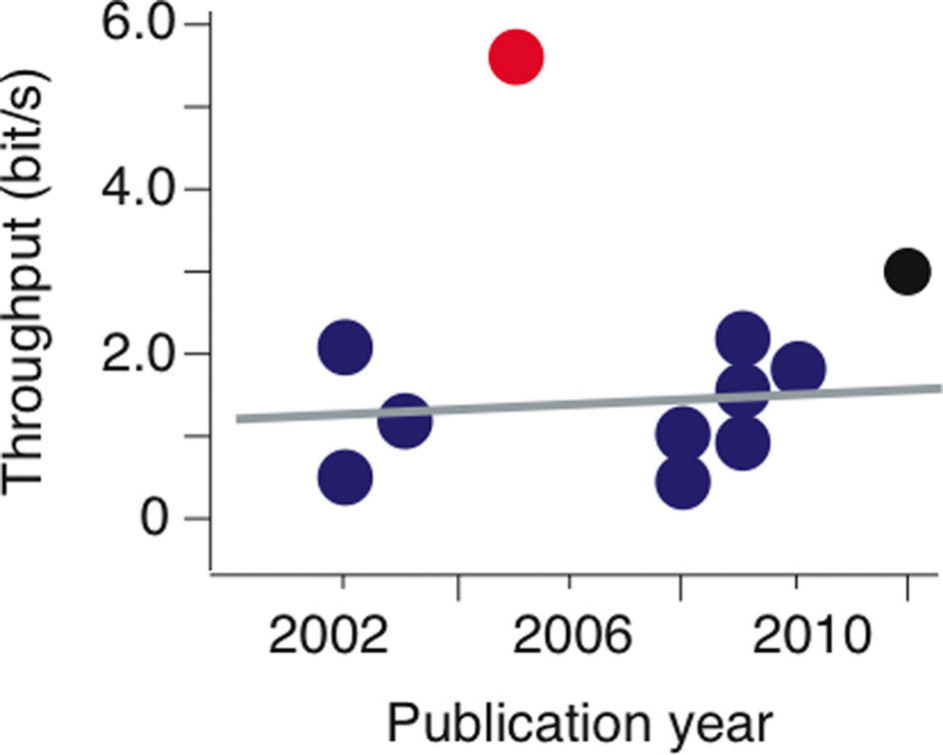
**Information transfer rates or throughput estimated for center-out reaching task (blue and black circles) and for “GO” cue task (red circle) in BMI papers.** Most data were taken from supplemental material of Gilja et al. (2012) while the “GO” cue data are for Santhanam et al. (2006) study. The line represents a linear fit for all blue data points; there was no significant increase in information transfer rates in this time period.

In invasive devices typically >20 of electrodes are used to pick up extracellularly brain signals (Schwartz et al., 2006, 2001). Usually, these brain signals are the so-called “single units,” representing action potentials of one or few neurons (Harris et al., 2000). When more than one single-unit is present in the trace from one electrode, spike sorting procedure is employed for their separation (Lewicki, 1998). In some cases local field potentials and multi-unit signals are also used for BMIs, these signals represent large populations of neurons (Andersen et al., 2004; Buzsáki et al., 2012; Aggarwal et al., 2013). How much information can be present in such recordings and how does the recording technology limit this amount of information?

It is much debated how much information a single action potential encodes. In visual system information rates up to ~1 bit/spike and >10 bit/s have been reported (Buracas et al., 1998). If all recorded single neurons were independent with no correlation between their action potential firing times, 10 neurons would correspond to 10 independent information channels and suffice to achieve information transfer rates of >100 bit/s (10 × 10). This rate is many orders of magnitude higher than typical rates of 1–2 bit/s achieved in invasive BMIs (Figure 2). In visual system neurons seem to be rather independent in information coding (Ecker et al., 2010), suggesting that potentially the information transfer rates that can be achieved with tens of recorded neurons in invasive BMIs is huge, well above the required 10 bits/s for fluent interaction with humans (see above) and magnitudes of order higher than currently achieved rates. One explanation for low information transfer rates achieved in current invasive BMIs could be that in motor systems only relatively few neurons encode large amounts of information and correlation between neurons is sufficiently high to reduce the amount of information encoded by the whole neuronal population (Carmena et al., 2003; Lebedev et al., 2008; Ecker et al., 2010). This inference is supported by the fact that for BMI control the pooled single unit information content is not much higher than the one found in the local field potential and multiunit signals, which are presumably the averages of large neuronal populations and individual differences of neurons are canceled out (Buzsáki et al., 2012; Aggarwal et al., 2013). In fact one of the best BMI performances in the terms of information transfer rates was achieved with no spike sorting, i.e., no neuron separation at each single electrode was used (Gilja et al., 2012).

One may argue that, since spike sorting procedure requires relatively high signal-to-noise ratio (Lewicki, 1998), low signal-to-noise ratio obtained with electrode arrays may result in poorly-separated single units, containing action potentials from more than one neuron, and the real independence of single neurons will be masked by such a contaminated single neuron signal (Ecker et al., 2010). However, in electrode arrays each recording site is sufficiently distant (>150 μm) from the other recording sites to ensure that no action potentials from the same neuron will be recorded by two recordings sites (Henze et al., 2000). Nevertheless, in electrode arrays correlation has been observed even for single units from different recording sites, corresponding to distinct, non-overlapping neuronal signals, thus it is unlikely that signal quality can account for relatively strong correlation between single units in motor system (Nicolelis and Lebedev, 2009; Ifft et al., 2013). These correlations between single units changed during learning to perform motor tasks, a clear indication that they are genuine and related to the motor control (Ifft et al., 2013). Finally, one of the best BMI performances in the terms of information transfer rates was achieved with no spike sorting, indicating that recorded single unit signal quality was not a limiting factor (Gilja et al., 2012). Thus it can be concluded that it is unlikely that insufficient quality of the electrode signal or amplification and filtration of the electrode signal limits the performance of current BMIs.

Another limitation of current technology is the number of simultaneously supported channels, usually it is the number of electrodes employed and the number of signal channels amplified and processed. The largest current systems can support up to 256 independent channels (Blackrock Microsystems, Salt Lake City, USA) while the largest electrode arrays may contain up to 96 electrodes. In one of the first papers on invasive BMIs it has been claimed that an almost unlimited accuracy of limb movement predictions could be achieved by increasing the number of recorded neurons (Wessberg et al., 2000). It was estimated that 400–500 of simultaneously recorded neurons would permit to achieve 90% accuracy. However, as the number of electrodes and simultaneously recorded neurons increased, the obtained data suggest no dramatic improvement beyond the first 40–100 neurons (Carmena et al., 2003; supplementary material in Gilja et al., 2012). Recently, based on a more extensive dataset, a logarithmic dependence of BMI performance (measured as SNR, see above) on the number of recorded neurons has been proposed (Lebedev and Nicolelis, 2011). It can be shown that essentially it is the same relationship suggested previously (Wessberg et al., 2000). It is asserted that with >10,000 of neurons the BMI performance would be on par with native hand (Lebedev and Nicolelis, 2011). Obviously the final proof of this claim will be an experiment with >10,000 recorded neurons. For now, several cautionary remarks can be made. The log/log scale is deceiving when a relatively narrow range of values is used, less than two orders of magnitude for recorded neuron numbers (from 2–3 to <200) and less than one order for SNR (from 0 to ~8 in 10^*^log_10_ scale, corresponding to 1–6.3, Figure 2 in Lebedev and Nicolelis, 2011); we still need to see if the same relationship holds for additional >2 orders for neuronal numbers and ~2 orders for SNR. Second, the graph is based on data tracking monkey walking, it is a stereotyped repetitive movement and it is not clear if the same relationship will hold for voluntary movements. One more note on the neuronal numbers regards neuron selection process in planar electrode arrays that are employed in most invasive BMIs of today. Since in such an array all electrodes are permanently fixed to the same platform it is impossible to adjust the penetration depth individually resulting in suboptimal location of electrode tips. It has been claimed that this is a serious issue that may compromise dramatically the BMI performance (Mulliken et al., 2008). In this paper it is shown that a better selection of <10 neurons in BMI with a flexible array with movable electrodes permits to achieve the same or even better performance than in a BMI with a rigid array and 10 times more recorded single units (~80). Nevertheless, the achieved performance is not better than reported previously; and probably it is safe to say that at least for the foreseeable future we will not achieve significantly higher information transfer rates by employing much larger arrays if no other changes are introduced.

The final note in support of the statement that currently technology is not a limiting factor in achieving higher information transfer rates in invasive BMIs is the absence of a clear increase in the information transfer rates in the last 10 years although the computer technology and electronics improved significantly, there were also advances in the electrode technology.

Clearly, this analysis on how the recording technology limits the amount of information present in the traces of extracellular electrodes is very brief. Nevertheless, it should be sufficient to state that electrode design, signal amplification and filtration, spike detection and sorting is unlikely to limit the information content present in these traces and there is a hope that the information content could be increased if the number of electrodes is increased >10 times. Then we have to accept the fact that in invasive BMI papers the recorded neurons probably encode only the reported amount of information about the predicted movement parameters. This conclusion is based in part on the fact that, for a given pair of parameters, let's say the neuronal spike rate and the cursor coordinate x, the information theory provides a precise formula to calculate the mutual information which is directly related to the maximal ability to estimate the value of one parameter when we know the value of the second parameter; in our example it would be the ability of the neuronal spike rate to predict the cursor coordinate x (Nelken and Chechik, 2007). Most algorithms that are used for movement control such as Kalman filters are known to efficiently utilize available information and it is unlikely that these algorithms dramatically limit the performance of BMIs. An example of an algorithm that does improve BMI performance nearly two-fold provided here below (Gilja et al., 2012) is largely based on a change of the movement parameters that are used for prediction but not the filters itself, the filters in the study were essentially a modified version of Kalman filter used by others also. We should remember that the information content is always calculated for a certain set of parameters that we want to predict and the information theory does not provide a recipe which parameters we should choose. It is possible in the same neuronal data set to find larger information quantity encoded by neurons if another movement parameter is chosen. To give an example, motor neurons may encode both the velocity and the position of a hand and but more velocity information is usually found in these neurons (Kim et al., 2011; Simeral et al., 2011). Thus, it is crucial to determine behavioral task or movement parameters for which the information quantity encoded by neurons is the highest. Essentially it is equivalent to asking what information the recorded neurons encode.

The presence of significant correlation between the measured parameter and the recorded neuronal activity does not mean that the recorded neuronal population is involved in determination of that parameter; correlation alone does not prove the presence of a causal link (Cramer, 2003; MacKinnon et al., 2007; Guilford and Fruchter, 1973). Actually in one of the papers considered to be a forerunner of the BMI concept (Fetz and Finocchio, 1971; Mussa-Ivaldi and Miller, 2003) the authors were asking very similar question: if we see a correlation between neuronal activity and muscle activity and movements, do these neurons participate in the generation of these movements? Since it was possible to force some neurons to be active when no muscle activity was present, even though just minutes before their activity correlated with the muscle activity, the answer was that correlation alone is not sufficient for such a claim.

BMIs may offer a new way to explore this issue. Intuitively it seems that the most efficient BMI will employ for the device control those parameters that are associated with the highest information content in the recorded neurons; this notion is confirmed by the information theory (Nelken and Chechik, 2007). Below two examples will be discussed to demonstrate how an unorthodox choice of parameters used for prediction enabled a dramatic increase in the information transfer rates in BMI.

One such an example has been reported by Shenoy group for the standard center-out reaching task (Gilja et al., 2012). Although in this case, similarly to the previous BMIs employing the center-out reaching task, the population of recorded neurons predicted the continuous movement parameters such as cursor position and velocity, there was a change in the coordinate system. Instead of trying to predict the cursor velocity with the respect to the observer, in other words instead of using a coordinate system fixed to the observer, it was assumed that the monkey always aims to the target, thus the coordinate axis was rotating toward the target as the cursor moved on. Although this modification was used only to predict cursor movements during algorithm training with brain control, the key fact is that such a modification of training introduced a coordinate system transformation for the velocity estimates. This modification alone significantly improved the information transfer rate in this task by <1 bit/s (less than two-fold). Although a relatively modest improvement, it was sufficient to achieve the best performance in this type of task (black circle in Figure 2) and to bring the target reach time to the range similar of the native hand (Gilja et al., 2012). A possible critique of this particular paper can be that apparently no limb fixation was used. In a number of studies it has been shown that there is a decrease in BMI performance following limb fixation (reviewed in Tehovnik et al., 2013). Nevertheless, it should be noted that Gilja and his colleagues demonstrated an improvement in BMI performance that resulted following the introduction of the new algorithm while all other conditions including hand fixation remained unchanged. Hence, the modification of coordinate system for algorithm training was sufficient to improve BMI performance by almost two fold. In addition, the same algorithm was used in an ALS patient with very limited hand movements and the achieved information transfer rate was well above the reported rates for similar patients (Henderson et al., 2013).

In invasive BMIs based on electrode arrays implanted in monkeys, the maximal reported information transfer rate was obtained in a 2006 study performed by the same Shenoy group (red circle in Figure 2, Santhanam et al., 2006). In contrast to all previous and most subsequent invasive BMI embodiments in monkeys, in this BMI the recorded neuronal population did not predict a parameter related to the continuous movement of a cursor such as velocity or coordinates, instead the recorded neuronal population was used to directly predict which target was chosen by the monkey. In the task employed in the study a monkey had to choose a limited number of targets following the “GO” cue and the algorithm predicted which target was chosen, no attempt to identify the movement trajectory was made. This change alone permitted to increase the information transfer rate from typical 0.8–1.5 bit/s to >6 bit/s. Such a >4 bit/s increase corresponds to more than one order increase in the information content. Although care was taken to have no eye movements during the period used to predict which target was chosen, it is clear that the signal used in this study is related to vision- the monkey had first to see the location of the target and then in one way or another this target location in the visual field of the monkey was reflected in the activity of neurons in the pre-motor area of the cortex.

This approach is somehow similar to the one taken in one of the most efficient forms of non-invasive BMIs in the terms of information transfer rates- row-column flickering based spellers (Wolpaw et al., 2002; Krusienski et al., 2008). In these spellers a subject watches a matrix of letters and symbols on a computer screen and each column or row increases in intensity for a brief period of time, 60–100 ms. Following 3–15 repetitions an accuracy >90% can be achieved. In non-invasive BMIs such an approach can routinely achieve information transfer rates of 0.25–0.5 bit/s, only two times less than it is achieved in standard center-out reaching tasks of invasive BMIs (Figure 2). Similarly to (Santhanam et al., 2006); most spellers use a vision related signal, either steady state evoked potentials (SSVEPs, reviewed in Vialatte et al., 2010) or P300, which in case of the EEG-based spellers is likely to be related to gaze (Brunner et al., 2010) and is clearly triggered by a visual signal. In spite of this similarity in the origins of the control signal, assuming that the “GO” cue task in Santhanam et al. (2006) and the speller tasks are similar, the invasive BMI is a clear winner with information transfer rates higher by >5 bit/s. This difference (>6 bit/s and 0.5 bit/s) correspond to a >~40 fold increase in the information transfer rates and only 3–4 bit/s shy of the rates typical for cochlear implants, ~10 bit/s. Thus, although the signal used by Santhanam and his colleagues is somehow similar to the non-invasive, EEG-based BMI signals P300 and SSVEP (Wolpaw et al., 2002), there is a dramatic difference in information transfer rates between invasive and non-invasive BMIs. May be we are not that far from electrode-array based invasive BMIs suitable for clinical applications?

The last example of an efficient invasive BMI presented here (Santhanam et al., 2006) suggests that the type of information encoded by the recorded neuronal signals in the cortical pre-motor areas is actually very different from what we usually think it is. This cortical area was used by many invasive BMIs although primary motor cortex may better predict upper limb movements (Wessberg et al., 2000; Carmena et al., 2003). It is difficult to compare information content in reaching and grasping tasks because we need to know the statistics of movements with and without BMI control, correlation coefficients alone cannot be directly translated into information content and, as explained above, the new suggested measure SNR (Li et al., 2009; Fitzsimmons et al., 2009) has not been validated experimentally. Although intuitively it may seem that, because of the 3-dimensionality and many degrees of freedom involved, 3D tasks correspond to large information transfer rates, Fitts' and subsequent studies on information content of human reaching movements suggest that a simple ratio of the target size (determined by the variability of the hand movement at the end point) to the distance to the target defines the information content of these movements (Fitts, 1954; MacKenzie and Buxton, 1992). Thus even for 3D tasks the information transfer rates may be similar to the ones present in 1D and 2D tasks, the data on which are shown in Figure 2.

If in the 3D-tasks the achieved information transfer rates are similar to the 2D-tasks, few bits per second, then may be these pre-motor neurons encode something that is not directly related to the movement trajectory? At least one study suggests that the answer is yes. It is clear that in Santhanam et al. (2006), study the pre-motor cortex neuronal signal that predicted targets was related to vision even though eyes were fixed during the period used for prediction; this choice of signal permitted to achieve the information transfer rates higher by >3–4 bit/s or by one order of magnitude compared to BMIs predicting movement. If such high information transfer rates can be achieved for a vision-related signal, maybe there is something fundamental that we are missing from the motor signal in primary and pre-motor areas? The “GO” cue permitted synchronization of all neuronal responses and a very brief time window of 250 ms could be used for decoding (Santhanam et al., 2006), something that is impossible to apply for a continuous movement decoding. It has been shown that the first few hundreds of milliseconds contain most information about the movement in the center-out reaching task (Taylor et al., 2003), indicating that after these first moments something is changed. The results of Gilja et al. (2012), suggest that the trick could be the changing coordinate system, in other words neurons encode in a coordinate system that is not fixed with respect to the subject's body/torso but moves together with the hand approaching the target.

It should be noted that, at least in theory, there is an entirely different method to achieve high information transfer rates, at least when we deal with very few neurons. It is the so-called conditional modulation that was used in 70 s (Fetz and Finocchio, 1971; Schmidt et al., 1978). It has been shown that rates up to >1 bit/s can be achieved with a single neuron (in an 8-target task the average time to target was 1.35 s, 97.5% correct, p. 359 in Schmidt et al., 1978). Similar approach was used by Kennedy and his colleagues when a single electrode was implanted in a human patient though much lower information transfer rates were achieved (<0.5 bit/s, Kennedy and Bakay, 1998). In a direct test of this idea in BMI the information transfer rates of 0.2–0.5 bit/s were achieved (Moritz and Fetz, 2011). A further modification of this approach is to directly stimulate muscles by a signal generated by such modulated neuronal activity (Moritz et al., 2008). The main question for this method is, can several neurons or groups of neurons be simultaneously modulated in independent fashion? It is known that monkeys are capable to voluntary modify the firing rates of neighboring neurons in opposite direction (Fetz and Baker, 1973); however it has yet to be shown if the same principle can be applied in BMI with at least 5–10 neurons. Nevertheless, potentially, such a method could offer very high information transfer rates even with a limited number of channels offered by current recording technologies.

As a final note, it should be added that there are new directions in BMI research that are difficult to evaluate from the point of view of information transfer rates. One such an example can be the recent demonstration that electrical stimulation of sensory areas may provide a sensation of a surface texture (O'Doherty et al., 2011). The difficulty of the task was low- the target size was almost the same as the distance between the targets and the estimated Fitts' difficulty of the task is <1.5 bit. For the achieved travel times between targets >1 s, the estimated information transfer rate was <1.5 bit/s, no improvement over typical rates in the center-out reaching task. However, this analysis misses the fact that in some case, due to brain stimulation, a monkey was able to determine if the target touched by the monkey was correct in less than <0.35 s. Even though it is only a yes or no decision and errors were present, the estimated information transfer rate is >2.5 bit/s. In addition one has to take into account that stimulation was performed in packets at 10 Hz, thus in reality the information transfer rates of >3 bit/s could be present. Furthermore, the texture sensation may be extremely valid for human patients to improve their comfort level with new BMI devices that would make this new technology more acceptable for the patients. The information transfer rates alone cannot be used to evaluate the performance of BMI also in an entirely new trend in BMI research- neuronal restoration (Grosse-Wentrup et al., 2011). Nevertheless, for device control information transfer rates offer a universal measure of BMI performance and may prove to be very useful in better understanding of our brain function.

In summary, the provided examples suggest that we still don't have sufficient understanding about what is encoded in premotor and motor areas of the cortex in order to achieve high rates of information transfer in invasive BMIs. Probably more effort should be devoted to understanding what kind of information is represented in recorded neuronal populations while technology issues seem to be secondary, at least for now. Research on invasive BMIs provides an unparalleled opportunity to test hypothesis on how movements are encoded in the brain. It seems that neurons in cortical pre-motor areas process a significant component of vision- or/and intention-related information that can be used to better predict movements; probably future BMIs should take into account this type of information. It is known that visual feedback is important for accurate hand movements (Saunders and Knill, 2003); in all invasive BMIs subjects can see the position of a cursor or a device. However, during decoding of neuronal signals vision-related or intention-related information is almost never used for invasive BMI control; probably, because it seems to contradict the idea of BMI, namely to predict movements from neuronal activity without other additional information. Since by far the best result in the center-out or similar tasks was achieved in an invasive BMI that does use intention- or/and vision- related information, maybe it is an indication that our brains never tries to predict limb coordinates alone but always combines different types of information and these different types of information are processed by neurons in cortical pre-motor areas and used to reach the desired target. There is nothing new in this idea (Hoshi and Tanji, 2004) and it should be no surprise that the use of intention or vision-related information permitted to Santhanam et al. to achieved maximal rate of 6 bit/s in invasive BMI, not much worse than it is achieved in natural movements, ~10 bit/s, thus assuring us that one day we will have prosthetic arms, controlled by brain almost as efficiently as we can control our own hands.

## Conflict of interest statement

The author declares that the research was conducted in the absence of any commercial or financial relationships that could be construed as a potential conflict of interest.
